# INTERGENERATIONAL HEALTH PROMOTION PROJECTS WITH COLLEGE STUDENTS

**DOI:** 10.1093/geroni/igad104.2066

**Published:** 2023-12-21

## Abstract

Presenters in this session describe intergenerational projects that bring college students, older adults, and others together to foster health and well-being.

